# 'A human face and voice': transgender patient-educator and medical student perspectives on gender-diversity teaching

**DOI:** 10.1186/s12909-023-04591-9

**Published:** 2023-09-01

**Authors:** Ky Ruprecht, William Dunlop, Estee Wah, Christine Phillips, Sarah Martin

**Affiliations:** 1 Medical School, Australian National University, Canberra, ACT 2600 Australia; 2 School of Medicine and Psychology, Australian National University, Canberra, ACT 2600 Australia; 3 Canberra Sexual Health Centre, Canberra Health Services, Yamba Drive, Garran, ACT 2605 Australia

**Keywords:** Transgender and gender diverse, Patient-educator, Small-group teaching, Contact theory, Curriculum development, Safe communication, Medical education

## Abstract

**Background:**

Transgender and gender diverse (TGD) people face many obstacles in accessing health care, including discrimination, institutional bias, and clinician knowledge deficits. We developed a clinical skills and education module on gender-affirming care for pre-clinical medical students, in collaboration with a TGD-led civil society organisation. The module consisted of an educational session followed by preceptor-facilitated small group tutorials, led by TGD patient-educators (*n* = 22) who used their lived experience to explore medical history-taking and broader issues related to TGD healthcare with students (*n* = 199). This study aimed to explore the views of students and TGD patient-educators on the structure, delivery and impact of the module.

**Methods:**

Analysis of responses of TGD patient-educators and students to the module (2020 and 2021), in post-intervention surveys using open-ended questions for TGD patient-educators (18 responses from 22 educators) and free text comments as part of a quantitative survey for medical students (89 responses).

**Results:**

Responses from students and patient-educators to the session were highly positive. Students and patient-educators emphasised that the teaching session succeeded through elevating the centrality of shared experience and creating a safe space for learning and teaching. Safety was experienced by patient-educators through the recognition of their own expertise in a medical environment, while students reported a non-judgemental teaching space which allowed them to explore and redress recognised limitations in knowledge and skill. Patient-educators described their motivation to teach as being driven by a sense of responsibility to their community. Preceptor attitudes may function as a barrier to the effectiveness of this teaching, and further attention should be paid to supporting the education of clinical facilitators in TGD health.

**Conclusion:**

The experiences of TGD patient-educators and medical students in this study suggest that this model of teaching could serve as a transferable template for TGD health and the inclusion of other historically marginalised groups in medical education.

**Supplementary Information:**

The online version contains supplementary material available at 10.1186/s12909-023-04591-9.

## Introduction

Transgender and gender diverse (TGD) individuals face challenges accessing healthcare and experience inequities in health outcomes compared to non-TGD people [1]. These disparities arise through the stigmatisation of TGD identities evidenced through overt prejudice, subtle discrimination, or implicit bias within medicine, often resulting in delayed presentations, avoidance of health services, poorer treatment or refusal should care be sought [2–4]. Personal bias and knowledge gaps among healthcare providers perpetuate adverse health outcomes for this population [2, 5, 6]. TGD medical students and health practitioners report experiencing direct bias from their peers [7], and many continue to keep their identity hidden [8].

Despite TGD people being recognised as medically vulnerable, training at all levels of medical education remains sub-optimal [3, 6]. Inclusion of TGD health in medical curricula has been positioned as a strategy to redress health disparities shouldered by this community [6]. Irrespective of the type of intervention, increased exposure to TGD-specific health has been shown to result in improved attitude and increased knowledge and/or skill caring for TGD patients; however, the impact on health outcomes for this population remains unclear [9–12].

Supervised contact between medical students and patients in structured clinical settings aims to develop empathy, challenge student bias and increase their understanding of the needs of medically underserved populations [13, 14]. Supporting TGD individuals as patient-educators to share their lived experience with medical students may help redress some of the health disparities that arise due to practitioner ignorance or bias [14–16]. The experience of patient-educators participating in medical education has been incompletely captured in the literature. Some studies explore the experience of patients participating in medical education in outpatient, inpatient [17, 18] and community [19] settings.. Rockey et al. explored the willingness and motivation of patients hospitalised in a tertiary hospital in the United States of America to participate in medical education; they concluded that most patients have an overwhelmingly positive experience and that motivation centres on a desire to help [17]. A mixed methods study of inpatient-educators from a tertiary Canadian hospital revealed similar themes. There was an overall positive attitude to medical student involvement in patient care, with patient-educators citing an opportunity to contribute to the education of others and in-depth discussion of their illness narrative as central themes to these encounters [18]. In a large systematic review of active patient involvement in medical education, one of the most cited was the desire among patient-educators to contribute to society and learning [19]. Preceptors often benefit from patient-educators, but tension can arise as to who is qualified to teach [20, 21].

Intergroup contact or contact-based education involves structured social interactions between individuals from different social groups. In medical education it has been positioned as a strategy to reduce stigma and discrimination against minority populations [22]. Few studies detail intergroup contact interventions between stigmatised or marginalised populations and health students. A randomised control trial by Pattern et al. that explored contact between patient-educators living with mental illness and pharmacy students concluded that students exposed to these contact-based sessions demonstrated a statistically significant reduction in stigma [23]. A similar intervention with 127 first-year medical students from the University of Valencia also concluded that brief, direct-contact interventions with patient-educators living with mental illness may serve to improve medical student attitude to this population [24].

We developed a clinical skills contact-based training module for pre-clinical medical students with TGD patient-educators, and demonstrated improvement in medical student attitudes and self-reported skill toward gender health care which was sustained at 1 month [25]. Here, we characterise and evaluate the experience of TGD patient-educators who participated in small-group learning as part of this module. This study aimed to explore the views of students and TGD patient-educators on the structure, delivery and impact of the module.

## Methods

The Medicinae ac Chirurgiae Doctoranda (MChD) is a four-year postgraduate degree offered by the Australian National University School of Medicine and Psychology (formerly Medical School). It consists of two pre-clinical years followed by two years of supervised clinical placements. Pre-clinical students have weekly clinical skills teaching with didactic presentations followed by supervised small group practice sessions with volunteer patients, usually delivered in face-to-face tutorials. These sessions cover a broad range of history-taking, examination, and procedural based skills. In 2020 and 2021, these sessions were delivered via an interactive online format in response to the COVID-19 pandemic. Prior to this intervention there was limited formal education in the pre-clinical curriculum on transgender health, with one scheduled lecture addressing the topic in passing. The motivation for curriculum reform was driven by informal feedback provided by successive cohorts of medical students who reported a knowledge gap in this field.

### Intervention

A new TGD clinical skills teaching module was developed by a sexual health specialist and academic with a clinical role in TGD healthcare (SM), a medical student (KR), and TGD educators and advocates from A Gender Agenda (AGA). AGA is a community-controlled civil society organisation for TGD and intersex people which provides peer support and advocacy for local and regional children, adolescents and adults. The module drew on an earlier project developing medical student competencies and learning outcomes, which KR had worked on in consultation with AGA staff and other TGD colleagues. Active feedback was sought from AGA and from patient-educators as to the content delivered during the lecture session and the format of the clinical skills session.. The module was introduced as a mandatory part of the curriculum. Content relating to TGD health care was delivered as an hour-long online lecture via Zoom with opportunities for students to ask questions throughout. Topics covered included terminology; principles of social, legal and medical affirmation; clinical guidelines and treatment pathways for TGD adults, adolescents and children; informed consent model of care; barriers and discrimination; preventative health care; principles of gender-affirming history taking; and physical examination and safety. Following the lecture students were given a short break before engaging in a one-hour tutorial (in small groups, via Zoom breakout rooms) designed to encourage students to practice gender-affirming history-taking and discuss TGD patient-educators’ experiences with the healthcare system. The learning objectives for lecture and tutorial are included in Appendix 3 and 4 respectively. Groups consisted of seven to nine students with a patient-educator, who could bring a support person if they chose. Tutorials were facilitated by clinical preceptors, all of whom were cisgender. Patient-educators were recruited through AGA by both word-of-mouth and their social media platforms. Patient-educators from the previous year were contacted by email and invited to participate again. Several patient-educators participated in both years of teaching. Online briefings were held for both preceptors and patient-educators prior to the tutorial session, and AGA facilitated a patient-educator debriefing. Briefings centred on structure of the tutorial session and discussion of the content; debriefing provided an opportunity to reflect on both positive and negative aspects of patient-educator involvement. AGA peer support was offered to all patient-educators in recognition of the potential harms of involvement in this teaching.

#### Roles

We used the term patient-educator to denote the roles of TGD volunteers who participated in the small group teaching through sharing their lived experience with students and participating in history-taking role play.

Clinical preceptors were doctors without specific training in TGD health and who regularly taught across the clinical skills program. The clinical preceptors were briefed on the module and agreed to be rostered. Their role in the tutorial was to facilitate discussions and contribute points of clinical practice if they became relevant to the conversation.

#### Data collection

Patient-educators were invited to reflect on and give written responses to questions about their motivations, expectations, and experiences of volunteering within a week of the teaching session. This study was approved by the ANU Human Research Ethics Committee (2020/236) and informed consent was obtained from participants involved in the study. Students were invited to respond to quantitative surveys described in Ruprecht et al. [25] before, one week, and one month after the teaching session. In the one-month survey, they were also asked to reflect on the teaching session. We included all responses even if students had not responded to other aspects of the quantitative arm of this study. A complete list of questions asked of patient-educators and students is available in Appendices 1 and 2.

#### Data analysis

Data for students and patient-educators were analysed separately using thematic coding. Authors read responses individually, then in a group, to familiarise themselves with the data. They then manually generated initial codes and refined them through cross-coding and joint reflection on the data, with a goal of achieving thematic inductive saturation [26]. The dataset was then analysed deductively for data saturation, to ensure adequacy of data collection [27]. The two sets of themes (students and patient-educators) were then cross-referenced and analysed together for cross-cutting themes. Patient-educator themes were: *Community responsibility as motivation to teach; Experience of teaching; Optimism.* Student themes included: *Perceived bias, Experience of learning, Gratitude.* Cross-cutting themes combining some of these themes included *Sharing a safe space* (experience of the session for both – combining *Experience of learning and Experience of teaching*)*,* and *the Centrality of lived experience* (using and recognising the expertise of patient-educators *– combining Gratitude and Experience of teaching*).

## Results

### Patient educators

Twenty-two patient-educators participated in small group teaching over the two years with four participating in both years. Ten of thirteen patient-educators gave feedback in 2020 and eight of thirteen in 2021; of the returning patient-educators, all four gave feedback in 2021. In total, eighteen responses were captured from the twenty-two patient-educators.

Demographic details of the patient-educator respondents are listed in Table 1. A random name generator was used to provide pseudonyms to patient-educators. The median age was 30 years, range 19–57 years.

**Table 1:** Demographic characteristics of TGD Patient-Educators

Patient-Educator	Age	Gender Identity	Participated 2020	Participated 2021
1. Kaylen	57	Non-Binary	Yes	No
2. Joe	28	Man	Yes	No
3. Elliott	19/20	Man	Yes	Yes
4. Savanna	43	Woman	Yes	No
5. Rory	31/32	Genderqueer/Non-binary	Yes	Yes
6. Louie	40	Man	Yes	No
7. Cooper	54/ 55	Man	Yes	Yes
8. Sam	23	Non-binary	Yes	No
9. Les	25	Transmasculine	Yes	No
10. Scout	49/ 50	Transmasculine	Yes	Yes
11. Wei	26	Non-Binary	No	Yes
12. Lorin	24	Genderfae	No	Yes
13. Sal	31	Man	No	Yes
14. Olly	29	Man	No	Yes

### Medical students

The combined cohort size was 199 students, of whom 89 provided feedback and 76 provided demographic data. All student respondents identified as cis-gendered, with 30 identifying as male and 46 as female. The median age of the medical students was 24, range 22–38 years.

#### Community responsibility as motivation to teach

The motivation for TGD patient-educators to teach was centred on improving the future health experiences of TGD people through the education of medical students. Many patient-educators detailed past negative experiences interacting with healthcare and a desire to improve health outcomes for other TGD people. Some patient-educators felt their story served as an archetypal TGD narrative, while others spoke of an obligation to contribute on behalf of other TGD people whom they perceived as potentially more vulnerable or less privileged than they were. Students’ motivation to learn was driven strongly by their understanding that they had many deficits in their knowledge for this patient population.

#### The centrality of lived experience: ‘I can bring a human face and voice’

During this teaching TGD patient-educators placed a key emphasis on TGD patients as experts in their bodies, the diversity of TGD experience, barriers to care, and understanding that TGD people have healthcare needs beyond gender-affirming care. Older TGD patient-educators placed particular importance on explaining the historical pathologisation of TGD identities to contextualise apprehension experienced by some TGD people when interacting with healthcare.

Patient-educators overwhelmingly felt that sharing their lived experiences could challenge student bias or ignorance and in doing so improve healthcare experiences for other TGD people. Many felt that by sharing their stories they were able to connect with students beyond stereotyped preconceptions of TGD persons. Some felt that through their involvement in this teaching they could learn more about themselves or the medical system.

Students identified an understanding of TGD health prior to this intervention and the value of the patient as an educator. They recognised the significant challenges faced by TGD people in this space and expressed a strong desire to learn how to provide healthcare to this population. Many students commented that they had never, to their knowledge, met or interacted with a TGD person and found hearing lived experiences in a small group setting invaluable. Others reflected on the limited exposure to TGD people in medical training. Students felt that by hearing TGD patient-educators’ perspectives they were able to see a ‘real person’ rather than an abstraction, which allowed for reflection on, and challenging of, their personal bias.

Students commented that the sharing of lived experience was complemented by the provision of up-to-date evidence-based resources. Students identified that the role of clinical preceptors during this teaching was to facilitate rather than educate, acknowledging the role of the TGD patient-educator as experts.

#### Shared safe space

Restrictions imposed by the COVID-19 pandemic meant online delivery of the 2020/21 sessions. We expected that a face-to-face teaching session could be a challenge for patient educators, but their reflections suggested that they would have preferred this to online communication with students. Patient-educators expressed an overwhelming desire to share physical space with students, commenting that the session would be improved by face-to-face teaching. Students also expressed a strong desire to have this session face-to face instead of online.

The importance of safety during this teaching was emphasised by TGD patient-educators. While TGD patient-educators identified altruism, empowerment, and increased understanding of self and medical education as benefits of participation, several acknowledged the emotional cost of participation and potential for harm. Some TGD patient-educators expressed surprise at the respectful engagement demonstrated by students during these teaching sessions, an indication that they were aware of the risks posed to their well-being by participating in this teaching. The value of discussion with people with lived experience was also noted by many students, with several showing insight as to the potential emotional toll upon, and generosity of, TGD patient-educators. The involvement of a peer-led TGD organisation in recruiting and supporting patient educator and positive experiences of other TGD people through word-of-mouth informed their assessment of safety. This aided recruitment for future sessions, with some patient-educators in 2021 citing this as a reason for participation. TGD patient-educators also highlighted the importance of pre- and post-teaching debriefing sessions to mitigate any distress caused to TGD patient-educators through the sharing of potentially traumatic experiences or negative reception in the teaching space from preceptors and/or students.

TGD patient-educators emphasised the importance of also creating a safe learning environment for students, allowing them the opportunity to ask questions, share their ideas, and challenge their own biases. TGD patient-educators also felt that learning was enhanced by a collaborative group discussion rather than single-interviewer history-taking. Students felt that small group teaching permitted a safe learning environment although many recognised this may be preceptor-dependent. Several students identified that preceptor and student bias could compromise the safety of TGD participants during this teaching session. Some medical students self-identified as queer, in their written feedback. In Australia ‘queer’ is used to define a range of genders and sexualities. These students expressed feeling obliged to contribute to creating a safe space by leading the discussion to demonstrate respectful engagement to other students. One student expressed concern that the students who may have gained the most from this session may have been silenced through this process. Non-queer identifying students may not have felt comfortable exposing their lack of knowledge; however even a silent witness may have benefitted through observing these interactions. When students were asked to comment on key messages taken from this teaching, respect emerged as a pervasive theme Tables 2, 3, 4, 5, 6 and 7.

**Table 2:** Community responsibility to teach

**TGD Patient-educator**
*‘I wanted to help current and future trans people have an easier time with the medical profession than I did.’ (*Kaylen*)*
*‘I feel obligated to share my experience to help students gain a better understanding of the issues faced by gender diverse people, and ultimately assist others in the community by doing so.’ (*Rory*, 2021)*
*‘… it gave me a sense that I was giving something of myself which was very useful to others both in how they shape themselves as medical professionals, but also potentially personally too.’ (*Louie*)*
*‘I am in a position to give my time and lived experience in this space and I see long term benefits in participation… it is important for the safety, health and vitality of our whole community.’ (*Scout*, 2021)*
**Medical student**
*‘Strengths were the willingness and obligingness of the volunteer patients to discuss their stories and provide insight.’*
*‘Speaking with the transgender patient was excellent. They gave us lots of insights into what it is like to be transgender and the issues that face them in accessing health care. The patient was so open and informative. I learned a lot.’*

**Table 3:** Centrality of lived experience

**TGD Patient-Educators**
*‘I can bring a human face and voice’ (*Scout*, 2021)*
*‘I believe that this kind of reflective practice is a parallel process to the provision of medical interventions to all patients but especially to TGD patients.’ (Sal)*
*‘Medical professionals may be the experts in their field, but we are the experts in our own bodies’ (Olly)*
*‘Maybe speaking to real humans and hearing real experiences will be a glimpse into what it’s like to navigate the medical system as a non-cis person who is just trying to access healthcare, rather than being a medical commodity to literally and metaphorically prod to satisfy the curiosity of medical professionals who feel entitled to ask invasive and inappropriate questions to non-cis patients.’ (Sal)*
*‘To help bring knowledge of being transgender to those it will help and maybe learn a bit about it all myself’ (*Savanna*)*
*‘I hope that students will have it personally reinforced that trans folk are just people, but also people that may have healthcare needs beyond being trans and transition-related care.’ (*Louie*)*
*‘The language we use to describe our bodies may in some cases differ from the textbook and so it’s best to take guidance from TGD people.’ (*Scout*, 2021)*
*‘Being older, it’s good to share the differences from being pathologised by the prevailing medical model to now being accepted.’ (*Cooper*, 2021)*
**Medical students**
*‘I think my opinions changed having learnt more and talked to a transgender person first-hand.’*
*‘I had previously been apprehensive about dealing with transgender people, not because I have anything against them, but because I didn’t really understand their perspective so was afraid of getting something wrong and offending them. It was great to be able to have that discussion in a safe environment without fear of causing offence.’*
*‘We are all human, and there are inequalities that I am unaware of…’*
*‘I think it is really important to have exposure to TGD people through medical training… I think it should make up a larger and more integrated part of the curriculum’*
*‘Before the session I didn’t really know how I would approach care for a transgender patient…’*

**Table 4:** Shared safe space

**TGD patient-educator**
*‘… this was my first ever time talking about the transgender aspects of my life. I hoped that the session would go well, that it would be safe, respectful and that I would be able to be empowered in the process.’ (*Scout*, 2020)*
*‘The students were really respectful, engaged and wanting to learn which was very heartening to see.’ (Joe)*
**Medical student**
*‘[A positive aspect of this teaching] was being able to ask questions in a safe space without fear of looking uninformed.’*
*‘Acceptance and respect are the most valuable features I can use in my practice.’*
*‘One of the things that our volunteer said to us is that it is okay to say you don’t know if we are uncertain about anything. The important thing is to listen and be understanding.’*

**Table 5:** Bias

**Medical student**
*‘My particular tutor had some, what I might consider, outdated views on trans health’*
*‘[Please] reiterate to tutors…keep your personal phobias to yourself.’*
*‘Our clinical skills tutors didn't have a lot of experience interacting with TGD people and I think that affected the dynamic a bit.’*
*‘I felt [this teaching] did not take into consideration other groups that have moral objections to transgenderism and did not give individuals who may be in those groups any good advice or strategies on working with trans individuals whilst they may have moral objections to their lifestyles.’*

**Table 6:** Prevailing optimism

*‘Honestly just the fact that this kind of session was run makes me more confident’ (Elliott)*
*‘The kids are alright’ (Rory, 2020) ‘Every session like this is a step in the right direction’ (Rory, 2021)*
*‘Even though they may never become an 'expert' in TGD healthcare, TGD people are everywhere and it's highly likely at some point in their careers they will have one as a patient.’ (Joe)*

**Table 7:** Improvements

**Patient-educator**
*‘Yeah maybe a bit longer. A chance for a follow up as discussed in the debrief session? A forum or I think another session with either the same group or a different would have its benefits.’ (Rory)* *‘Being face to face, I would love to be in the physical space’ (Wei)*
**Medical student**
*‘Perhaps the volunteers could swap rooms and we could have another session with a different person of the trans community in order to understand different experiences.’* *‘It sounded like each volunteer had a very different and interesting story. It might be good to have rotating rooms so students get to hear from multiple volunteers or format it as a panel discussion where each person can tell their story briefly and then questions at the end.’* *‘The session was excellent, some of the regular tutors could maybe receive more education before the session to prevent them misgendering patients but clearly that’s the weakness of the medical school not this session in particular.’*

#### Experience of bias in the teaching space 

Students observed that variability in preceptor attitude may impact the efficacy of this teaching and compromise volunteer safety and wellbeing and suggested that increased training or vetting tutors for problematic or harmful views may be appropriate for these teaching sessions. Students acknowledged that these clinical attitudes underlie the difficulties TGD people face when attempting to access healthcare. Students also identified that some preceptors had a knowledge gap with TGD health.

Despite overwhelmingly positive responses for this teaching, a small minority of students expressed attitudes that may cause intentional or inadvertent harm to the TGD community. No TGD patient-educator who provided feedback commented that they experienced hurtful comments during this teaching session, however, feedback surveys were not received from three patient-educators in 2020 and five in 2021.

#### Prevailing optimism

TGD patient-educators were asked if they felt their future medical needs were more likely to be met by students participating in this teaching and if they felt these students would be better able to provide care to TGD patients. Responses from patient-educators were overwhelmingly positive with varying degrees of confidence. Despite all respondents commenting on past poor treatment in healthcare settings, many felt that the increasing normalisation of gender diversity meant a reduction in stigma held by current medical students which will translate to improved care of TGD people in the future. Patient-educators recognised that students had variable familiarity and comfort with gender diversity and TGD health and emphasised the importance of providing baseline knowledge to students who will invariably treat a TGD patient in the future.

#### Improvements for the future

Patient-educators and medical students offered suggestions for future improvement for this teaching that centred on content, delivery, and safety. Both suggested there may be value in the TGD patient-educators delivering the pre-tutorial lecture in conjunction with the medical specialist. Many reinforced their preference of face-to -face teaching as opposed to online learning. Patient-educators suggested increased tutorial time and the option for a follow-up forum. Some students recognised the limitations of exposure to only one TGD patient-educator and called for improved diversity of representation during the tutorial. Others identified lack of specific preceptor training as a potential threat to TGD patient-educator safety during tutorials. Medical students also identified lack of perceived preparedness for the teaching and requested additional pre-session resources.

## Discussion

This paper describes the views of TGD patient-educators and students on a novel transgender health clinical skills program for pre-clinical students. TGD patient-educators in this study acknowledged that inadequate training of medical students and health practitioners underpinned poor health experiences of the TGD community which is consistent with the established literature [6, 28, 29]. Education of medical students as a mechanism to improve these health outcomes served as the core motivation for participation in this teaching. Student feedback for this teaching intervention was overwhelmingly positive, acknowledging a deficit in TGD health coverage and valuing the power of lived experience to challenge both pre-existing bias and knowledge gaps. The tutorial was perceived to be particularly valuable and there was support for its ongoing inclusion in the curriculum. The literature recognises several barriers to the inclusion of TGD teaching across all levels of medical education including perceived lack of importance of subject matter, inexperience of faculty to deliver the intervention, limitations of dedicated teaching time and challenges in recruiting and compensating transgender guest speakers [30–32]. Despite this, medical students’ consistently recognise the value of including training in the curriculum that serves diverse patient populations [33].

Students overwhelmingly cited deficits in knowledge about TGD health and gender diversity as the central barrier to the provision of appropriate care to this population rather than overt transphobia. This was also identified as the key barrier by medical residents in the United States [34]. The lack of inclusion of TGD health in the medical curriculum and the need for increased dedicated teaching in this area has also been reported in a survey of Canadian medical students [35].

The motivation of TGD patient-educators to participate in this teaching primarily centred on benefits to community and improving healthcare experiences and potentially outcomes of others [6]. Increased understanding of medical education and oneself as well as empowerment were also perceived benefits [36]. The centrality of lived-experience and patient contact was consistently echoed by TGD patient-educators as a potential mechanism to reduce stigma through normalisation and increased empathetic understanding of challenges specific to TGD patients. This strategy has been utilised in medical education with other historically-stigmatised populations, such as persons living with HIV or mental illness [37].

In medical education there remains limited consensus as to the most effective TGD teaching intervention with significant variability across institutions regarding content, delivery, and post-intervention effectiveness measures [6]. To our knowledge no other study in the literature examines transgender-patient educator experience in participating in medical education interventions. It is now widely acknowledged that including patients’ lived experience improves health provider understanding of challenges unique to their condition; this is particularly important for historically marginalised populations [38]. Irrespective of session format, placing TGD people at the centre of teaching remains essential to the development of appropriate educational content and respectful delivery [39]. This was supported by our patient-educators who consistently reiterated the importance of including TGD people in clinical skills teaching demonstrating obligation and willingness to be involved in transgender health education.

The primary challenge identified through this study for TGD patient-educator participation in medical student teaching was concerns that re-living traumatic experiences may negatively impact their wellbeing. Some patient-educators acknowledged that they did experience stress, and felt that while they were able to cope, some TGD people may be more vulnerable to the negative aspects of participation. Peer-led recruitment and post-teaching session debriefing were highlighted as important mechanisms to ensure volunteer safety during these interventions. Cultivating a strong relationship with local TGD community organisations is another key to collaborative, respectful, and safe teaching in this space [40].

Students recognised that preceptor bias, in the form of both overt transphobia or subtle microaggressions (intentional or unintentional slights that communicate hostile, derogatory or negative attitudes towards TGD individuals), potentially affected student attitude and compromised the safety of TGD patient-educators. While studies consistently identify lack of provider knowledge as a barrier to care for TGD patients, Stroumsa et al*.* concluded that the presence of transphobia amongst primary care providers rather than hours of education dictated provider competence in this sphere [3]. This has been hypothesised to reflect the historical pathologisation of transgender identities within medicine where a binary gender system which is congruent with biological sex is reinforced [41, 42]. This overarching social framework, which is considered morally and biologically correct, establishes normal and abnormal ways of being, with individuals who deviate from this order subject to socially condoned scrutiny [43, 44]. Medical students, through the hidden curriculum, absorb and model their clinical preceptors through tacit transfer of subjective values which, if negative, may undermine formal teaching of TGD health [45]. A small number of students expressed attitudes that may cause intentional or inadvertent harm to TGD community, supporting the assertion that bias and transphobia may be highly resistant to change [3]. Students participating in this teaching were pre-clinical, therefore, consideration must be given to the influence of continued exposure to the informal and hidden curriculum of medical education during clinical years in shaping attitudes to TGD health that they carry into their future practice.

An area of improvement elicited from both patient-educator and student feedback was the content delivered before the tutorial sessions. While students recognised the value of a lecture given by a physician with an academic and clinical interest in TGD health, some students suggested that the session may be improved by including TGD patients during the lecture presentation. This was echoed by some patient-educators who also felt that they should participate in the pre-tutorial lecture. Some students commented that the lecture content was too general and introductory while other students appreciated that no assumptions were made about the baseline knowledge across the cohort. Other students felt that the option for pre-lecture question submission or anonymous questions during the lecture would be a valuable addition to this teaching.

Student feedback highlighted student preparedness. Though most students felt they learned a great deal from the exploration of patient-educator’s lived experience, some expressed a desire for a more structured process and clearer instruction on interacting with the patient-educator. This may reflect discomfort with this interactive pedagogical style which challenges students to reflect upon their own biases. To address unevenness in pre-session student knowledge, development and delivery of an introductory online learning module to complete before the clinical skills day could be utilised [46].

Another area that warrants further examination is the shared safe space, and how we might create this in more deliberate and mindful ways. While there is a recognised need for this teaching in collaboration with TGD people there is also the potential to cause harm to patient-educators, and the onus falls onto medical schools and clinical preceptors to ensure safe spaces for these interactions.

We are cognisant of limitations to this study. We have no feedback from eight of the twenty-two patient-educators (36%), meaning negative or poor experiences may have gone uncaptured. There is also a marked absence of transfeminine and female voices in patient-educator feedback. While patient-educators in this study described students as enthusiastic and respectful, we must allow that student participation in this teaching may have been affected by perceived social desirability, with those who held negative views perhaps being less likely to interact during teaching or provide post-intervention feedback [47]. Ensuring students have insight into the impacts of intersectionality and exploring intersectionality of patient-educator experience would add a rich discussion to future research in this space. While our study aimed for diversity of experience of TGD patient-educators, we were grateful for all individuals who volunteered their time and did not pursue interrogation of individual social class, ethnicity, sexuality, religion or disability status.

## Conclusion

This education module demonstrates the value of working respectfully with patient-educators who can share their expertise and lived experience. The collaborative development and delivery of this teaching intervention with a local TGD community organisation helped support the safety of patient-educators and the session itself honoured their experience. Medical students noted the humanising role of TGD patient-educators in increasing their knowledge of barriers to healthcare access. Preceptor attitudes may function as a potential barrier to the efficacy of this teaching, and further attention should be focused on supporting the education of these clinician-facilitators in areas they are unfamiliar. The experiences of TGD patient-educators and medical students in this study suggest that this model of teaching could serve as a transferable template for TGD health or the inclusion of other historically marginalised groups in medical education at other teaching institutions. Medical schools wishing to do this should consider appropriate payment for patient-educators, preceptor selection and preparation, and collaborative development, delivery and evaluation of curricular materials.

### Supplementary Information

**Additional file 1: Appendix 1.** Questions asked of patient-educators. **Appendix 2.** Questions asked of student participants. **Appendix 3.** Learning objectives for the lecture. **Appendix 4.** Learning objectives for the tutorial.

## Data Availability

The datasets generated during the current study are not publicly available due to the small number of participants providing personally sensitive qualitative data. Data are available from the corresponding author on reasonable request.

## Abbreviations

TGD: Transgender and Gender Diverse
AGA: A Gender Agenda

## References

[1] Cheung AS, Ooi O, Leemaqz S, Cundill P, Silberstein N, Bretherton I (2018). sociodemographic and clinical characteristics of transgender adults in Australia. Transgend Health.

[2] Samuels EA, Tape C, Garber N, Bowman S, Choo EK (2018). "Sometimes you feel like the freak show": aq qualitative assessment of emergency care experiences among transgender and gender-nonconforming patients. Ann Emerg Med.

[3] Stroumsa D, Shires DA, Richardson CR, Jaffee KD, Woodford MR (2019). Transphobia rather than education predicts provider knowledge of transgender health care. Med Educ.

[4] White Hughto JM, Reisner SL, Pachankis JE (2015). Transgender stigma and health: a critical review of stigma determinants, mechanisms, and interventions. Soc Sci Med.

[5] Vermeir E, Jackson LA, Marshall EG (2018). Barriers to primary and emergency healthcare for trans adults. Cult Health Sex.

[6] Dubin SN, Nolan IT, Streed CG, Greene RE, Radix AE, Morrison SD (2018). Transgender health care: improving medical students' and residents' training and awareness. Adv Med Educ Pract.

[7] Freeman NW, Keuroghlian AS (2022). The art of medicine: supporting transgender and gender diverse medical students. Lancet.

[8] Dimant OE, Cook TE, Greene RE, Radix AE (2019). Experiences of transgender and gender nonbinary medical students and physicians. Transgend Health.

[9] Park JA, Safer JD (2018). Clinical exposure to transgender medicine improves students' preparedness above levels seen with didactic teaching alone: a key addition to the Boston University Model for Teaching Transgender Healthcare. Transgend Health.

[10] Dowshen N, Nguyen GT, Gilbert K, Feiler A, Margo KL (2014). Improving transgender health education for future doctors. Am J Public Health.

[11] Braun HM, Garcia-Grossman IR, Quiñones-Rivera A, Deutsch MB (2017). Outcome and impact evaluation of a transgender health course for health profession students. LGBT Health.

[12] Eriksson SES, Safer JD (2016). Evidence-based curricular content improves student knowledge and changes attitudes towards transgender medicine. Endocr Pract.

[13] Burgess A, van Diggele C, Roberts C, Mellis C (2020). Key tips for teaching in the clinical setting. BMC Med Educ.

[14] Batt-Rawden SA, Chisolm MS, Anton B, Flickinger TE (2013). Teaching empathy to medical students: an updated, systematic review. Acad Med.

[15] Towle A, Bainbridge L, Godolphin W, Katz A, Kline C, Lown B (2010). Active patient involvement in the education of health professionals: active patient involvement in education. Med Educ.

[16] Walch SE, Sinkkanen KA, Swain EM, Francisco J, Breaux CA, Sjoberg MD (2012). Using intergroup contact theory to reduce stigma against transgender individuals: impact of a transgender speaker panel presentation: using intergroup contact theory to reduce stigma. J Appl Soc Psychol.

[17] Rockey NG, Ramos GP, Romanski S, Bierle D, Bartlett M, Halland M (2020). Patient participation in medical student teaching: a survey of hospital patients. BMC Med Educ.

[18] Elfassy MD, Duncan LJ, Green A, Sun H, Guimond T, Tzanetos K (2020). Patients as teachers: Evaluating the experiences of volunteer inpatients during medical student clinical skills training. Can Med Educ.

[19] Dijk SW, Duijzer EJ, Wienold M (2020). Role of active patient involvement in undergraduate medical education: a systematic review. BMJ Open.

[20] Wykurz G, Kelly D (2002). Developing the role of patients as teachers: literature review. BMJ (Clin Res Ed).

[21] Sharma M (2018). ‘Can the patient speak?’: postcolonialism and patient involvement in undergraduate and postgraduate medical education. Med Educ.

[22] Crystal DS, Killen M, Ruck M (2008). It is who you know that counts: intergroup contact and judgments about race-based exclusion. Br J Dev Psychol.

[23] Patten SB, Remillard A, Phillips L, Modgill G, Szeto ACH, Kassam A (2012). Effectiveness of contact-based education for reducing mental illness-related stigma in pharmacy students. BMC Med Educ.

[24] Atienza-Carbonell B, Hernández-Évole H, Balanzá-Martínez V (2022). A "patient as educator" intervention: reducing stigmatizing attitudes toward mental illness among medical students. Front Public Health.

[25] Ruprecht K, Dunlop WA, Wah E, Phillips C, Martin SJ. Intergroup contact improves medical student attitudes and skill in transgender healthcare. TransgendHealth. 2023 epub ahead of print 23 May 2023. 10.1089/trgh.2021.0203. 10.1089/trgh.2021.0203 PMC10998020 38585241

[26] Braun V, Clarke V (2006). Using thematic analysis in psychology. Qual Res Psychol.

[27] Saunders B, Sim J, Kingstone T, Baker S, Waterfield J, Bartlam B (2017). Saturation in qualitative research: exploring its conceptualization and operationalization. Qual Quant.

[28] Obedin-Maliver J, Goldsmith ES, Stewart L, White W, Tran E, Brenman S (2011). Lesbian, gay, bisexual, and transgender-related content in undergraduate medical education. JAMA.

[29] Sanchez AA, Southgate E, Rogers G, Duvivier RJ (2017). Inclusion of Lesbian, Gay, Bisexual, transgender, queer, and intersex health in Australian and New Zealand medical education. LGBT Health.

[30] Gamble Blakey A, Treharne GJ (2019). Overcoming Barriers to Transgender Healthcare Education in Aotearoa New Zealand. N Z J Educ Stud.

[31] Teal CR, Gill AC, Green AR, Crandall S (2012). Helping medical learners recognise and manage unconscious bias toward certain patient groups. Med Educ.

[32] van Heesewijk J, Kent A, van de Grift TC, Harleman A, Muntinga M (2022). Transgender health content in medical education: a theory-guided systematic review of current training practices and implementation barriers & facilitators. Adv Health Sci Educ Theory Pract.

[33] Verbree A-R, Isik U, Janssen J, Dilaver G (2023). Inclusion and diversity within medical education: a focus group study of students’ experiences. BMC Med Educ.

[34] Johnston CD, Shearer LS (2017). Internal medicine resident attitudes, prior education, comfort, and knowledge regarding delivering comprehensive primary care to transgender patients. Transgend Health.

[35] Chan B, Skocylas R, Safer JD (2016). Gaps in transgender medicine content identified among Canadian Medical School Curricula. Transgend Health.

[36] Lauckner H, Doucet S, Wells S (2012). Patients as educators: the challenges and benefits of sharing experiences with students: Patients as educators. Med Educ..

[37] Jaworsky D, Gardner S, Thorne JG, Sharma M, McNaughton N, Paddock S (2017). The role of people living with HIV as patient instructors – reducing stigma and improving interest around HIV care among medical students. AIDS Care.

[38] Chu LF, Utengen A, Kadry B, Kucharski SE, Campos H, Crockett J (2016). "Nothing about us without us"-patient partnership in medical conferences. BMJ.

[39] Arora M, Walker K, Luu J, Duvivier RJ, Dune T, Wynne K (2020). Education of the medical profession to facilitate delivery of transgender health care in an Australian health district. Aust J Prim Health.

[40] Noonan EJ, Sawning S, Combs R, Weingartner LA, Martin LJ, Jones VF (2018). Engaging the transgender community to improve medical education and prioritize healthcare initiatives. Teach Learn Med.

[41] Carrera-Fernández MV, Almeida A, Cid-Fernández XM, Vallejo-Medina P, Rodríguez-Castro Y (2020). Patrolling the boundaries of gender: beliefs, attitudes and behaviors toward trans and gender diverse people in Portuguese adolescents. Int J Sex Health.

[42] Worthen MGF (2016). Hetero-cis-normativity and the gendering of transphobia. Int J Transgend.

[43] Murphy M (2014). Hiding in plain sight: the production of heteronormativity in medical education. J Contemp Ethnogr.

[44] Bauer GHR, Travers R, Kaay M, Hohenadel K, Boyce M (2009). “I don't think this is theoretical; this is our lives”: how erasure impacts health care for transgender people. J Assoc Nurses AIDS Care.

[45] Cribb A, Bignold S (1999). Towards the reflexive medical school: The hidden curriculum and medical education research. Stud High Educ.

[46] Gacita A, Gargus E, Uchida T, Garcia P, Macken M, Seul L (2017). Introduction to safe space training: interactive module for promoting a safe space learning environment for LGBT medical Students. MedEdPORTAL.

[47] van de Mortel TF (2008). Faking it: social desirability response bias in self-report research. Aust J Adv Nurs.

