# Supplementary material for: 'A human face and voice': transgender patient-educator and medical student perspectives on gender-diversity teaching
Source: BMC Med Educ. 2023 Sep 1;23:621. doi: 10.1186/s12909-023-04591-9 (PMC10472656; doi:10.1186/s12909-023-04591-9)
Supplement: Supplementary file 1 — Additional file 1: Appendix 1. Questions asked of patient-educators. Appendix 2. Questions asked of student participants. Appendix 3. Learning objectives for the lecture. Appendix 4. Learning objectives for the tutorial. [file 12909_2023_4591_MOESM1_ESM.pdf]

*Appendix 1.* Questions asked of patient-educators.

1. Is this the first time you have volunteered as a patient for medical students?
2. What motivated you to participate?
3. What did you hope to get from this session?
4. What do you hope the students will get out of this session?
5. What was the most positive aspect of this experience?
6. What was the most negative aspect of this experience?
7. How could the session be improved?
8. Would you participate in a session like this again? Why, or why not?
9. These students will become doctors in 2023/24. Do you feel more confident, or less confident, that your future medical needs would be met by students participating in this teaching session? Why, or why not?
10. Do you feel these students will be better able to provide care to the TGD patients after participating in this session Why, or why not?

*Appendix 2.* Questions asked of student participants.

1. What did you think the strengths/benefits of the session were?
2. What did you think were the weaknesses of the session; or areas that could be improved?
3. What was the key message you took from the session?
4. Do you have any suggestions for improving the session?

*Appendix 3.* Learning objectives for the lecture.

- Create safe, inclusive and affirming environments for trans and gender diverse (TGD) patients
- Analyse key issues for wellbeing and understand barriers that exist for TGD people accessing transition and non-transition related health
- Identify and use a patient's name and pronouns
- Use a sensitive approach to taking gender-affirming and non-transition related medical histories
- Discuss considerations for performing safe physical examinations for TGD patients
- Analyse the preventive care needs for TGD patients
- Analyse the needs of parents, carers and family of trans children, adolescents or adults
- Assess supports for trans people and their families

*Appendix 4.* Learning objectives for the tutorial.

| ANU Medical School TCH Campus | **Phase 1 CLINICAL SKILLS** |
|---|---|

## Guide to Key Steps: Transgender and Gender Diverse Care

**KSG for taking a history of gender identity, gender affirming care and other medical history from trans and gender diverse patients.**

**Resources:** Patient (or parent of a patient) identifying as transgender or gender diverse.

**Example Introduction:** *"We are in general practice. This patient has come to discuss issues related to transgender or gender diverse care. I would like you to take a history, being aware of the sensitivities surrounding gender dysphoria, gender affirmation and ongoing care as outlined in the KSG'.*

| ***Key Step Guide*** |
|---|
| Welcome, thank patient for volunteering today; gain consent for the interview. |
| Identify and use chosen name and pronouns and promote their use by other others in your group; appreciate that sex assigned at birth may differ from gender identity. |
| Invite the patient's thoughts on what it means to be transgender. |
| Discuss the psychological impact of gender dysphoria, if any, and assess the impact of gender affirmation/transition on the patient's emotional wellbeing/mental health. |
| Sensitively discuss the patient's transition care. Recognise that transition is different for everybody. |
| Determine the nature of any current treatment plan. |
| Specifically obtain detail regarding a patient's hormone treatment or intention to undertake hormone treatment. |
| Enquire about gender affirming surgery or plans for surgery. |
| Ask about other medical history. |
| Obtain details of health professionals involved in the patient's care. |
| Discuss the process and costs involved in accessing hormone replacement therapy and surgery. |
| Discuss the process of social and legal transition including coming out; documentation (birth certificates/name change/sex marker); impact on employment, family and relationships. |
| Discuss any positive or negative experiences with healthcare professionals relating to the patient's gender identity. |
| Determine any barriers experienced by the patient when accessing medical treatment. |
| Assess the impact of gender affirmation/transition on the patient's physical, emotional or social life including family, relationships and employment. |
| Determine the extent to which the patient's overall lifestyle promotes health. |
| Enquire about past screening (prostate/cervical) for a patient's relevant anatomy. |
| Discuss support services available to TGD patients and their families. |
